# A Pilot Study of British and Irish Junior Doctors in Auckland, New Zealand, Using a Questionnaire to Explore Motivations and Intentions

**DOI:** 10.7759/cureus.96413

**Published:** 2025-11-09

**Authors:** Katherine Makris, Yvonne Chan, Christopher Lewis

**Affiliations:** 1 General Medicine, Auckland City Hospital, Auckland, NZL; 2 Medical Education, Auckland City Hospital, Auckland, NZL; 3 Respiratory Medicine, Auckland City Hospital, Auckland, NZL

**Keywords:** junior doctor training, national health service (nhs), new zealand, physician recruitment, physician satisfaction

## Abstract

Introduction

As dissatisfaction grows amongst junior doctors working in the NHS and Health Service Executive (HSE, Ireland’s national public health system), an increasing number of them are reported to be considering leaving the UK and Ireland. Over the last few decades, New Zealand has been an emigration destination for many doctors. This preliminary study of British and Irish junior doctors working in Auckland, New Zealand, aimed to explore their motivations for leaving the UK and Ireland and discover if their expectations had matched their experiences, in order to aid further research across the country.

Methods

An online survey was sent to all British and Irish house officer doctors working in public hospitals in the Auckland region in March 2025. Responses were analysed with descriptive statistics.

Results

The survey was sent to 55 doctors and there were 23 responses. Many reported dissatisfaction with working life, more so in the NHS and HSE than Te Whatu Ora (New Zealand's national public health system). Twenty-one out of 23 (91%) respondents had moved to New Zealand seeking a better work-life balance, however some felt they had less free time working in Te Whatu Ora. Nineteen out of 23 respondents (83%) intended to leave New Zealand, mainly to be closer to family and friends.

Conclusion

Despite most of this cohort of British and Irish junior doctors moving to New Zealand for a better work-life balance, some felt they worked more hours in Te Whatu Ora. Most still intended to return home, regardless of potential changes in Te Whatu Ora. Further research across the country is needed to determine if these findings are representative of all British and Irish junior doctors in New Zealand.

## Introduction

In recent years, it has been well publicised in the media and in research that British junior doctors (known as resident doctors in the UK) are leaving or planning to leave the NHS [1-4]. In 2023, a survey of UK medical students found that a third planned to leave the NHS within two years of graduating [5]. In 2022, a survey of British junior doctors found that one-third planned to leave the NHS to work abroad in the following year, with most planning to move to Australia and New Zealand [1]. Similar trends have been found in the Health Service Executive (HSE, Ireland’s national public health system), with one study finding that Ireland had the highest percentage of doctors working abroad of all countries in the European Union and the UK [6]. With increasing dissatisfaction and disillusionment with the NHS in the UK [2] and HSE in Ireland [7], it is possible that the percentage of junior doctors wanting to leave the NHS and HSE is even greater now.

Compared to other the Organisation for Economic Co-operation and Development (OECD) countries, New Zealand employs the highest proportion of international medical graduates [8]. New Zealand has often been a destination for British and Irish doctors to move to [1,2,9,10]. Despite this, there is little recent evidence investigating the motivations for junior doctors who have moved to work in New Zealand, and their thoughts about returning to work in the NHS and HSE.

This preliminary study evaluated junior doctors who had completed their foundation training (the first two years post-graduate medical training) in the NHS or intern year (the first year post-graduate medical training) in the HSE and had moved in the following few years to work for Te Whatu Ora (New Zealand’s national public health system). The aim was to explore the factors leading to their leaving the NHS or HSE, and their subsequent experience compared to their expectations. Their intention to return to the NHS or HSE was also explored. This pilot study was based in Auckland as it is New Zealand’s most populous city and has several public hospitals in the region. As a preliminary study, the findings can be beneficial to provide insights and areas of interest for further research of a larger cohort more widely across the country, which could be of use to the NHS, HSE, and Te Whatu Ora for the purpose of workforce planning.

## Materials and methods

All the British and Irish junior doctors working at a house officer level in the hospitals in the Auckland region in March 2025 were invited to an online survey via email. The list of doctors was compiled by the Clinical Education and Training Unit (CETU) at Auckland City Hospital and included all British and Irish house officer doctors working in a public hospital in the Auckland region in March 2025. These individuals were identified due to the national medical council requirements for supervision, which is organised through CETU. The inclusion criteria: (1) those currently working in the Auckland region at house officer level, (2) those who had completed medical school in the UK or Ireland, and (3) those who had completed the first two years of post-graduate work in the UK or the first year of post-graduate work in Ireland. The exclusion criteria was those who had been working as a doctor for more than five years. All those who completed the questionnaire were deemed eligible by this criteria. It is recognised that this method of convenience sampling risks the cohort being unrepresentative of the population, however this method was felt to be sufficient to generate initial ideas and opinions of the sample, which could be explored in more detail in further research.

The email invite to the survey was distributed by CETU to prevent email addresses being disclosed to the researchers. The survey remained open for three weeks in March 2025 with two reminders sent a week apart. Participants could complete the survey anonymously. The project was discussed with the research ethics department at Auckland City Hospital and formal ethical approval was deemed to not be required. Participation in the study was voluntary, however, those who completed the survey were invited to join a random draw to win a 100NZD gift card as an incentive.

Most of the questions were multiple-choice to ease respondent burden and increase uptake. The multiple-choice answers included those that were used in previous studies of more senior UK-trained doctors working in New Zealand, in addition to the themes that were elicited from ‘other’ responses in those studies [9,11]. Given the similarities in the cohort of this study to the previous studies, it was felt that these multiple-choice options could provide initial selection options, however an ‘other’ option with free text was given in most questions for the respondent to detail other opinions. 

The survey was piloted and reviewed with members of the CETU team, who work closely with British and Irish house officers, and have experience in surveying the target population. Suggestions to the survey were given by individuals and amalgamated until all the reviewers were satisfied with the survey. 

The questions were structured into three parts: experiences working in the NHS and HSE, experiences working in Te Whatu Ora, and the intention to return. To compare the satisfaction of working in the NHS and HSE to working in Te Whatu Ora, participants were asked to score their satisfaction with their working life and also with the amount of time and energy they were left with after work for family, social and recreational activities on a scale of one to 10. A score of one indicated very unsatisfied and 10 was very satisfied. This way of measuring satisfaction was used in the 2015 study with NHS-trained doctors working in New Zealand [11]. Cronbach’s alpha was used to assess the internal consistency of satisfaction with working life and satisfaction with time and energy after work to determine if there was a relationship between these and thus consistency in overall satisfaction.

The data was analysed with descriptive statistics, including percentages and mean averages. Further statistical analysis as well as analysis of subgroups was not performed in view of the small number in the cohort.

## Results

Demographics

The survey was sent to 55 junior doctors. There were 23 responses, giving a response rate of 42%. Table 1 summarises the demographics of the survey’s respondents.

**Table 1:** Demographics of the survey’s respondents Values are represented by number (and percentage)

Parameter	Number (and percentage) of respondents
Gender		
Woman	16 (70%)
Man	7 (30%)
Age		
20-24	1 (4%)
25-29	18 (79%)
30-34	4 (17%)
Ethnicity		
White (incl. English, Welsh, Scottish, Northern Irish and Irish)	16 (70%)
Asian or Asian-British	4 (17%)
Mixed or multiple ethnic groups	3 (13%)
Number of years working as a doctor		
2	2 (9%)
3	13 (57%)
4	7 (30%)
5 or more	1 (4%)
Time spent working in New Zealand		
<3 months	5 (22%)
3-6 months	6 (26%)
6-9 months	5 (22%)
9-12 months	3 (13%)
1-2 years	4 (17%)

The ethnicity options given were the same as those used in the most recent UK census [12].

Experience working in the NHS and HSE

The mean satisfaction score for working life in the NHS and HSE was 5.56, with the mean satisfaction score with the amount of time and energy after work for family, social and recreational activities being even lower at 4.35. Cronbach’s alpha for these two scores was 0.70, indicating a moderately correlated relationship and thus a similar underlying thoughts on overall satisfaction.

**Figure 1:**
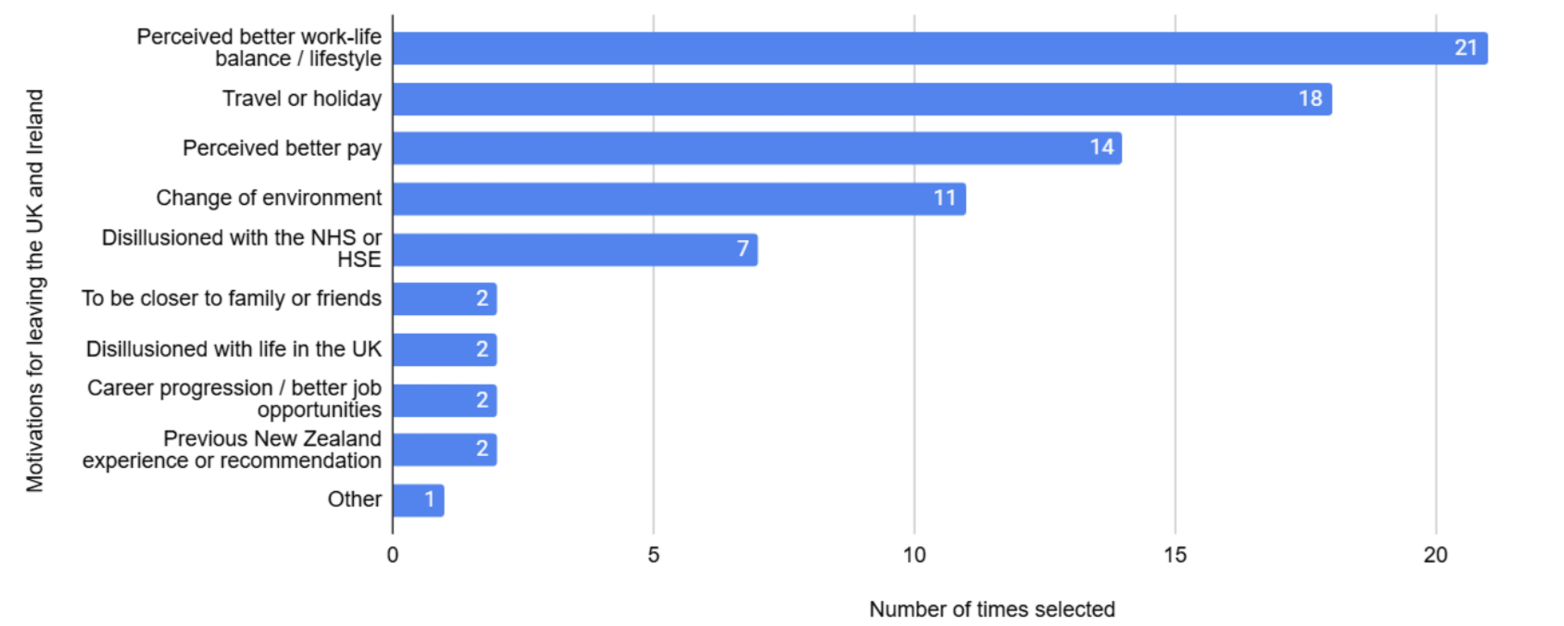
Selected motivations for leaving the UK and Ireland

When asked to rank the top three motivations for leaving the UK and Ireland out of a pre-selected list, only 17/23 (74%) completed this correctly, with multiple respondents selecting more than three options. Perceived better work-life balance or lifestyle was chosen as the top motivation in nearly half of those answering this question correctly (8/17 (47%)), with all the remaining participants still ranking this in their top three motivations. In the whole cohort, 21/23 (91%) ranked perceived better work-life balance or lifestyle as a motivation for leaving the UK. Figure 1 shows how many times each answer was selected by all the respondents.

One respondent selected other but did not expand on this further.

When asked how long they had intended to stay in New Zealand prior to leaving the UK or Ireland, 16/23 (70%) intended to stay for one to five years in New Zealand, 6/23 (26%) stated less than one year, and 1/23 (4%) intended to stay permanently in New Zealand. One participant, who intended to stay for less than a year, had been in New Zealand for one to two years. No other respondents of this survey had yet spent the amount of time they intended to stay in New Zealand. This is likely because this project was looking at junior doctors within the first few years of moving to New Zealand.

Experiences working in Te Whatu Ora

The mean satisfaction score of working life in Te Whatu Ora was 7.09, in comparison to a mean satisfaction score of 5.6 in the NHS and HSE. When asked about satisfaction with the amount of time and energy after work, the mean score was 6.13, in comparison to a mean satisfaction of 4.35 in the NHS and HSE. Cronbach’s alpha of the two New Zealand satisfaction scores was 0.92, indicating very strong relationship between the satisfaction scores.

Intention to return

**Figure 2:**
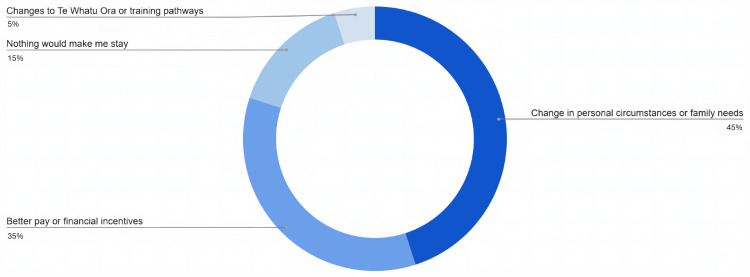
Selected options that would encourage those planning on returning to their home countries to stay in New Zealand Values are represented as percentage of participants.

Nineteen out of 23 respondents (83%) stated that they intended to return to the NHS or HSE (either ‘yes, definitely’ or ‘yes, probably’). Three out of 23 (13%) responded that they were not intending to return (either ‘no, definitely’ or ‘no, probably’). One respondent out of 23 (4%) was unsure. Of those that intended to return, when asked about their main motivation for returning, the vast majority (84%) selected to be closer to family and/or friends. One selected disillusionment with life in New Zealand. Two respondents selected the ‘other’ option, citing ‘training programme more supported (in the UK)’ and ‘career progression + to be closer to friends’ as their reasons. When asked if anything would encourage them to stay in New Zealand, the most selected option was personal circumstances and not work-related. Figure 2 shows the responses to this question.

Those who were not intending to return to work in the NHS or HSE, or were unsure, were asked if there were any factors that would change their decision. The four following options were each selected once: better working conditions; better pay or financial incentives; changes in personal circumstances or family needs; and, nothing would make me return. 

The final question was an optional free text for participants to share anything additional about their work experience or motivations. Table 2 shows the four verbatim responses. 

**Table 2:** Verbatim responses of additional information that participants volunteered about their work experiences or motivations alongside demographics

Participant demographics	Response
Woman, aged 25-29, 4 years post-graduate, 3-6 months in New Zealand	I like working here! Lots of opportunities to learn and develop. Relief rotas are quite gruelling, and 16-hour-long days are hard though.
Woman, aged 25-29, 3 years post-graduate, 3-6 months in New Zealand	Surprised by how much worse the rota was for general medicine here. In UK would work max 7 days (infrequent), here frequently working 10 days in a row which is exhausting and leaves little room for life outside of work. Also frequently having to stay late, which again leaves little time for life outside of work
Man, aged 30-34, 3 years post-graduate, <3 months in New Zealand	A better and more structured approach and orientation for UK (junior doctors) joining the workforce
Woman, aged 25-29, 3 years post-graduate, 1-2 years in New Zealand	In general very positive experience working in (New Zealand) - much more supportive and encouraging work environment with better work/life balance. Would love to stay longer and enter training programmes, but unfortunately too far from family - would require change in personal circumstances. But will definitely be recommending to other Irish doctors when I return home!

## Discussion

This study aimed to provide an initial understanding as to why British and Irish junior doctors chose to leave the UK and Ireland to work in New Zealand, and whether their experience has been a positive one. Whilst small, the findings provide a unique insight into junior doctors’ opinions of working in the NHS or HSE and Te Whatu Ora and their motivations for choosing to stay or return. This is particularly important at a time when the UK has large number of doctors moving to practice abroad [13].

This sample of junior doctors reported much dissatisfaction with the working life, in particular from their experiences in the NHS and HSE. The difference between satisfaction with working life in New Zealand compared to the UK has also been reported in previous studies, including more senior doctors, over the last few decades [9,14,15]. It has been suggested that some main reasons for doctors being more satisfied in New Zealand include systemic resilience by employing doctors to work in ‘relief positions’ to fill rota gaps during inevitable periods of doctor absence, better leave and funding for study and exams, and free meals during work to ensure doctors feel valued [15,16]. Additionally, due to the larger population with more urban living, there can be greater job stress in the UK compared to New Zealand leading to a less rewarding working life [14].

There is a paucity of research looking specifically at British or Irish doctors emigrating to New Zealand in recent years, however recent research on international medical graduates (IMGs) working in New Zealand highlighted that cultural differences, in particular with differences in communication as well as needing to understand cultural intricacies of typical Māori (New Zealand’s indigenous population) practices, were challenging and led to some considering leaving New Zealand [17,18]. But, as these studies looked at IMGs from many countries worldwide, it is not possible to know if these were the feelings of British and Irish doctors. One respondent in this cohort did comment on the “orientation for UK (doctors) joining the workforce”, which may have been mentioned due to need for orientation on cultural differences. However, there was no further detail to this comment to confirm this.

In 2012, Sharma et al. found that the mean satisfaction score for working life amongst doctors in New Zealand was 8.1 [9], compared to this study where the mean satisfaction score in New Zealand in 2025 was 7.09. The 2012 study also found that the mean satisfaction with time and energy after work was 7.8 [9], compared to this study’s mean satisfaction score of 6.13. These findings could suggest that doctors are more unhappy with their working life now compared to thirteen years ago, importantly with the COVID pandemic happening within this time. Findings from the USA found that, in the first years of the pandemic, a large proportion of doctors intended to reduce their clinic workload due to increased burnout and decreased work fulfilment [19]. In migration terms, Irish doctors working abroad returned to Ireland earlier than planned due to the pandemic, citing wanting to help their home country and to be closer to family. Some doctors reported that COVID clarified the importance of being closer to family and friends [20]. This has been echoed in research in Poland, which found that whilst COVID itself did not influence migration intentions of doctors, the pandemic highlighted other ‘push’ and ‘pull’ factors, such as being closer to family, that impacted migration decisions [21]. The cohort in this study would all have been either in the final years at medical school or first years of working as a doctor through the pandemic. Further research is needed to uncover whether the pandemic or changes in global mobility affected their decisions to move to New Zealand or consider returning.

Nearly all participants chose to leave the NHS and HSE due to a perceived better work-life balance or lifestyle, or for travel or holiday. This has been seen in similar research, with “pull” factors of better working conditions and lifestyle being found to drive doctors to New Zealand [9,11,22]. Despite this, two participants commented on the longer hours worked in New Zealand, “leav(ing) little room for life outside of work”, suggesting that these junior doctors’ experiences had possibly not met their expectations. However, another participant who had been working in New Zealand for longer suggested that Te Whatu Ora allowed for a “better work/life balance”. A literature review of 40 studies looking at doctor migration in and out of the UK found that better employment opportunities and training programmes were cited as reasons to leave the UK [22], however no participants in this study selected the option of career progression or better job opportunities as a motivation for moving to New Zealand.

Only one participant in this study considered staying in New Zealand permanently when leaving the UK and Ireland. Other studies have found similarly low percentages of doctors intending to stay in New Zealand when they left the UK [8,9]. Having worked in Te Whatu Ora, most still intended to return to their home country, primarily to be closer to family and friends, highlighted by one participant responding that New Zealand is “unfortunately too far from family”. A large part of this cohort stated that only changes in personal circumstances would make them stay in New Zealand, regardless of any potential changes in Te Whatu Ora.

One participant had also given the reason that the “training programme (is) more supported” in the UK. Improved training opportunities have previously shown to influence doctors’ decision to stay or leave New Zealand [9]. Pay and wage options were also frequently chosen throughout the survey. Perceived better pay was ranked highly as a reason for leaving the UK and better pay or financial incentives was chosen by many as a reason that would encourage doctors intending to leave, to stay. This could suggest that pay and financial incentives were not as favourable as the junior doctors had expected.

Limitations

Whilst these findings highlight new information about the British and Irish junior doctors working in New Zealand, it is important to mention the limitations as there were inevitable biases associated with the methodology. The sample size of the study was small, with respondents being just less than half of all British and Irish house officers working in Auckland. The demographic differences between respondents and non-respondents was not measured and so non-response bias cannot be excluded, meaning the results may not be truly representative of the target population, particularly given the gender imbalance of the cohort. Selection bias may also may have been present due to the inclusion of a participation incentive. Furthermore, the results from dissatisfied doctors who may have already left New Zealand would not be included in this cohort, thereby potentially inflating the satisfaction scores in New Zealand. Retrospectively scoring satisfaction from the time in the UK or Ireland may also have led to inaccuracies in recalling satisfaction score in the NHS or HSE.

As the study was only conducted in Auckland, New Zealand’s largest city, similar research across other parts of New Zealand is needed to determine if the results are replicated in different regions, for example, in more rural settings. This would be particularly interesting as doctors are paid nationally standardised salaries as per the Single Employer Collective Agreement (SECA) [23]; however, Auckland’s cost of living is considerably higher than the rest of the country.

Furthermore, this study utilised a questionnaire with mostly multiple-choice answers. A study involving interviews or focus groups could allow for more novel ideas and enable a more in-depth qualitative analysis. As no statistical analysis was performed and confounders, including specialty and time spent working in New Zealand, were not accounted for, the results of this study are not representative of all British and Irish junior doctors working in New Zealand, however given the lack of recent information on this group, the study does provide preliminary findings to aid further large-scale research.

## Conclusions

This preliminary study of British and Irish junior doctors working in the Auckland region in March 2025 found that many were dissatisfied with their working life, more so in the NHS and HSE than Te Whatu Ora. Most intended to return to work in their home countries, with the vast majority wanting to be closer to family or friends. Whilst some felt that their decision to leave New Zealand could not be changed, financial incentives or more supported training programmes were offered as factors that could encourage some to stay. Given the biases in the methodology, these results are preliminary and further research is needed across New Zealand to determine if the findings are representative of the population as a whole.

## Appendices

Appendix A: 

**Table 3:** Questionnaire questions with possible multiple-choice responses

Question	Possible responses
How would you describe your gender?	Female
	Male
	Non-binary
	Prefer not to say
Please indicate your age range	20-24
	25-29
	30-34
	35-39
	40-44
	45+
	Prefer not to say
How would you describe your ethnicity?	White (including English, Welsh, Scottish, Northern Irish, Irish)
	Mixed or multiple ethnic groups
	Asian or Asian British
	Black, African, Caribbean or Black British
	Other ethnic group
	Prefer not to say
How many years have you worked as a doctor?	2
	3
	4
	5 or more
How long have you been working in New Zealand?	<3 months
	3-6 months
	6-9 months
	9-12 months
	1-2 years
	3-4 years
	5+ years
Which hospital are you currently working at?	Auckland City Hospital
	Starship Children’s Hospital
	Green Lane Clinical Centre
	North Shore Hospital
	Waitakere Hospital
	Middlemore Hospital
	Manukau Super Clinic
What specialty are you interested in working in in the future?	Internal medicine
	Surgery
	General practice / Urgent care
	Obstetrics and gynaecology
	Paediatrics
	Psychiatry
	Emergency Medicine
	Anaesthetics
	Intensive Care
	Radiology
	Public health
	Other
Prior to leaving the NHS, on a scale of 1-10 how satisfied were you with your working life in the NHS? (1=very unsatisfied, 10=very satisfied)	1 – 10 scale
Prior to leaving the NHS, on a scale of 1-10 how satisfied were you with the amount of time and energy you were left with after work for family, social and recreational? (1=very unsatisfied, 10=very satisfied)	1 – 10 scale
At the time of leaving, what were your motivations for leaving the UK? Rank your top 3 options	Career progression/better job opportunities
	Perceived better work-life balance/lifestyle
	Perceived better pay
	To be closer to family or friends
	Travel or holiday
	Disillusioned with the NHS or HSE
	Disillusioned with life in the UK
	Change of environment
	Previous New Zealand experience or recommendation
	Other (free text option)
Prior to leaving the UK, how long did you intend to stay in New Zealand?	<1 year
	1-5 years
	Long term / permanent
	Unsure
On a scale of 1-10, how satisfied are you with your working life in Te Whatu Ora and New Zealand? (1=very unsatisfied, 10=very satisfied)	1 – 10 scale
On a scale of 1-10 how satisfied are you with the amount of time and energy you are left with after work for family, social and recreational? (1=very unsatisfied, 10=very satisfied)	1 – 10 scale
Do you intend to return to work in the NHS in the UK?	Yes definitely
	Yes probably
	Unsure
	No probably
	No definitely
If answered yes to above, what is the main motivation for returning to work in the UK?	Career progression/better job opportunities
	Better work-life balance/lifestyle in the UK
	Better pay in the UK
	To be closer to family or friends
	Travel
	Disillusioned with Te Whatu Ora
	Disillusioned with life in NZ
	Preferred life in the UK
	Other (free text option)
If answered yes to above, would any of the following encourage you to stay in New Zealand?	Better working conditions
	Better pay or financial incentives
	Changes to Te Whatu Ora or training pathways
	Change in personal circumstances or family needs
	Nothing would make me stay
	Other
If answered no to above, would any of the following change your decision?	Better working conditions
	Changes to the NHS or training pathways
	Greater recognition of New Zealand qualifications
	Better pay or financial incentives
	Change in personal circumstances or family needs
	Nothing would make me return
	Other
Is there anything else you would like to add for this study to describe your experiences of work in the UK compared to New Zealand or your motivations for moving to New Zealand or wanting to stay in New Zealand or return to the UK?	Free text
